# Robust neutralization of SARS-CoV-2 variants including JN.1 and BA.2.87.1 by trivalent XBB vaccine-induced antibodies

**DOI:** 10.1038/s41392-024-01849-6

**Published:** 2024-05-09

**Authors:** Xun Wang, Shujun Jiang, Wentai Ma, Yanliang Zhang, Pengfei Wang

**Affiliations:** 1 Shanghai Pudong Hospital, Fudan University Pudong Medical Center, State Key Laboratory of Genetic Engineering, MOE Engineering Research Center of Gene Technology, School of Life Sciences, Shanghai Institute of Infectious Disease and Biosecurity, Fudan University, Shanghai, China; 2 Department of Infectious Diseases, Nanjing Hospital of Chinese Medicine Affiliated to Nanjing University of Chinese Medicine, Nanjing Research Center for Infectious Diseases of Integrated Traditional Chinese and Western Medicine, Nanjing, Jiangsu China; 3 Beijing Institute of Genomics, Chinese Academy of Sciences, University of Chinese Academy of Sciences and China National Center for Bioinformation, Beijing, China

**Keywords:** Vaccines, Immunology

**Dear Editor**,

The rapid and widespread transmission of SARS-CoV-2 has led to the emergence of multiple variants of concern (VOCs), most notably the Omicron variant, which continues to evolve and diversify into a range of sub-lineages. Our previous research has shown that these Omicron sub-lineages, spanning BA.1–BA.2.86, have been evolving to exhibit increased neutralization escape capabilities.^1–4^ Since November 2023, the JN.1 variant, stemming from BA.2.86’s antigenic diversity and acquiring an additional mutation (L455S) in RBD, has rapidly emerged as the dominant strain. Additionally, a newly identified, highly mutated SARS-CoV-2 variant, BA.2.87.1, was detected in South Africa between September and November 2023, which has recently been classified as a variant under monitoring (VUM) and has sparked global concern. While it originates from the ancestral BA.2 lineage, BA.2.87.1 is genetically distinct from the currently circulating Omicron lineages. It exhibits over 100 mutations, with more than 30 in the spike protein, including notable changes in the receptor binding domain (RBD) like K417T, K444N, V445G, and L452M, which are crucial for antibody recognition. Intriguingly, this variant has 7 fragment deletions, including 3 in the spike protein, with 2 of which encompass over 10 crucial amino acids (Del 15–26 and Del 136–146) in the N-terminal domain (NTD) (Fig. 1a). This evolutionary strategy, which involves sacrificing parts of the virus to evade the immune system, makes these deletions potentially more significant than nonsynonymous mutations. Given our existing immunity from vaccinations and past infection, the effectiveness of this immunity against BA.2.87.1 remains to be determined.

**Fig. 1:**
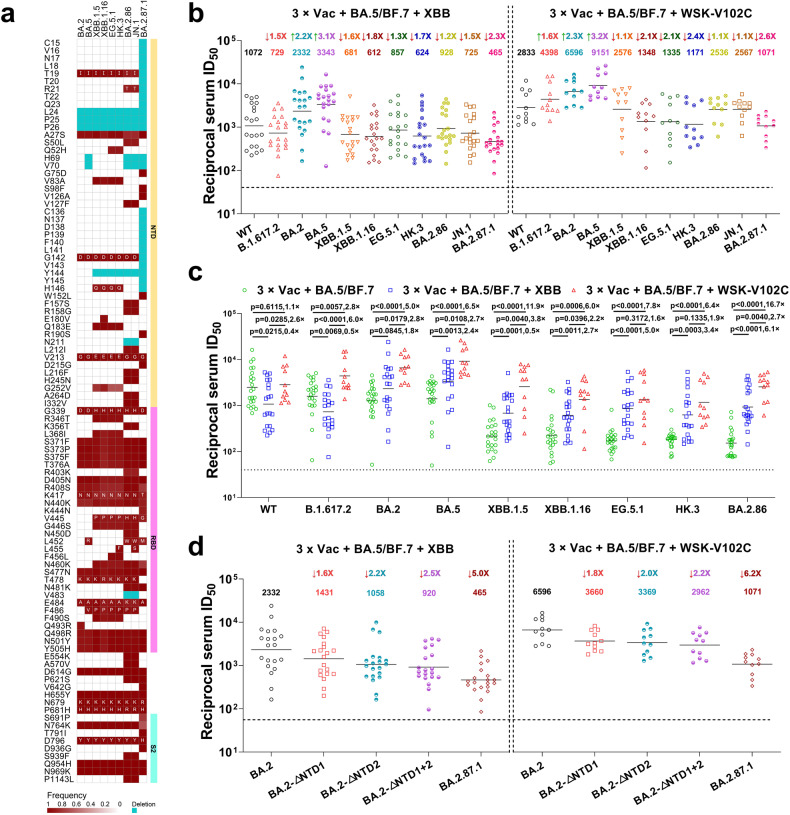
Neutralization of distinct SARS-CoV-2 sub-lineages by XBB reinfection and WSK-V102C vaccination sera. **a** The mutation frequency heatmap of BA.2.87.1 and other related lineages. Only mutations with a frequency higher than 0.75 are shown. Deletions are shown in cyan. Mutation frequency data was retrieved from the outbreak.info website. **b** Neutralization of different SARS-CoV-2 variant PsVs by sera collected from two groups of individuals who had previously experienced BA.5/BF.7 breakthrough infections following three doses of inactivated vaccines: one group were reinfection with XBB virus, the other group were boosted with the WSK-V102C vaccine. **c** In parallel comparison of neutralization GMTs against distinct Omicron subvariants by sera collected from individuals with XBB reinfection or with WSK-V102C booster vaccinations. **d** Neutralization of PsVs representing BA.2.87.1’s single and combined NTD deletions by sera collected from individuals with XBB reinfection or with WSK-V102C booster vaccination. Values above the symbols denote GMTs and their fold increase or decrease relative to WT (for panel **b**) or BA.2 (for panel **d**). Dotted lines indicate the threshold of detection (40 for all the cohorts). *P* values were determined by using multiple Mann–Whitney tests. WT wild-type

The swift rise of antigenically diverse SARS-CoV-2 variants and decreasing vaccine efficacy against infection have necessitated updates in COVID-19 vaccine formulations. In September 2023, the monovalent XBB.1.5 mRNA vaccine received approval from the United States Food and Drug Administration (FDA). Concurrently, China approved several XBB-adapted vaccines, including WestVac BioPharma’s Coviccine® Trivalent XBB Vaccine (WSK-V102C). This vaccine incorporates the RBDs of the XBB.1.5, BA.5, and Delta variants, fused with the spike protein’s heptad repeat (HR) domain and self-assembled into stable trimeric protein particles. It is further enhanced with a squalene-based oil-in-water emulsion adjuvant, added post-purification and mixing for increased efficacy.^5^

From December 2022 to January 2023, over 80% of China’s population experienced BA.5/BF.7 infections despite receiving three doses of inactivated vaccines. Subsequently, from May to July 2023, approximately one-fifth of the population was affected by the XBB wave. It is critical to assess whether the immunity in these subpopulations from XBB reinfection remains protective. For those uninfected by XBB, understanding how the efficacy of XBB-containing booster vaccines compares to natural immunity from breakthrough infection is vital, especially considering the emergence of new variants like JN.1 and BA.2.87.1.

In this study, we collected blood samples from two groups of individuals who had previously experienced BA.5/BF.7 breakthrough infections following three doses of inactivated vaccines. One group (*n* = 20) experienced XBB reinfection, while the other (*n* = 11) received the WSK-V102C vaccine. Samples were collected about 1-month post-infection or vaccination. To evaluate their serum neutralization potency and breadth, we employed a panel of SARS-CoV-2 pseudoviruses (PsVs), including the wildtype (WT), B.1.617.2, BA.2, BA.5, XBB.1.5, XBB.1.16, EG.5.1, HK.3, BA.2.86, JN.1, and BA.2.87.1. Our previous study revealed that individuals with BA.5/BF.7 breakthrough infections primarily had high neutralizing titers against WT, moderately reduced (~2-fold) against Delta and early Omicron variants, but significantly lower (~10-fold) against XBB sub-lineages and BA.2.86. Interestingly, sera from XBB reinfection displayed a shifted neutralization pattern, showing the highest geometric mean titer (GMT) of 3343 and 2232 against BA.5 and BA.2, respectively, i.e., about 2-3-fold higher than that against WT. However, these sera showed remarkably decreased titers against XBB descendant subvariants (e.g., XBB.1.5, XBB.1.16, EG.5.1, HK.3) as well as BA.2.86 and its descendant JN.1, approximately 1.2–1.8-fold lower than that against WT. Notably, the lowest titer was observed against the latest variant, BA.2.87.1, with a GMT below 500 (Fig. 1b).

In contrast, sera from the group having received the WSK-V102C vaccine exhibited markedly higher neutralizing titers against all the tested variants. The highest neutralization titers were observed against BA.5 (GMT = 9151), followed by BA.2 (GMT = 6596) and Delta (GMT = 4398). While these sera also demonstrated reduced titers against XBB descendant subvariants, BA.2.86, JN.1, and BA.2.87.1, they maintained higher neutralization titers compared to the sera from the XBB reinfection group, although BA.2.87.1 exhibited a higher escape potential than BA.2.86 and JN.1. Even so, the sera from individuals vaccinated with the WSK-V102C vaccine maintained a GMT above 1000 against BA.2.87.1 (Fig. 1b), suggesting that booster vaccination with WSK-V102C is expected to be effective in protecting individuals from BA.2.87.1 infection. Interestingly, we observed distinct antibody responses in the participants of the three groups: (1) those with BA.5/BF.7 breakthrough infections,^4^ (2) those with additional XBB reinfection, and (3) those vaccinated with WSK-V102C post-breakthrough infection. The XBB reinfection group showed recalled antibody responses against post-Omicron variants but less against pre-Omicron variants like WT and Delta. In contrast, the trivalent vaccine induced significantly more potent and broader neutralization activity (Fig. 1c). The elevated titers against Omicron variants may be attributed to the BA.5 and XBB.1.5 components in the vaccine, while the heightened response to Delta variants may be due to the vaccine’s Delta component.

For the BA.2.87.1 variant, the most notable mutations are likely the two fragment deletions in NTD. To assess their impact on neutralization sensitivity, we constructed PsVs with each of these fragment deletions and a combination of both based on the spike protein of the BA.2 variant. The sera from individuals with XBB reinfection demonstrated a 1.6-fold reduction in neutralizing titer against the BA.2-ΔNTD1 (Del 15–26) PsV and a 2.2-fold reduction against BA.2-ΔNTD2 (Del 136–146) PsV, compared to the parental BA.2 PsV. Additionally, the PsV-carrying spike protein with both fragment deletions (BA.2-ΔNTD1 + 2) showed an even further reduced sensitivity, at 2.5-fold lower than BA.2 PsV. A similar pattern was observed with sera from the individuals having received the WSK-V102C vaccine, indicating that both fragment deletions in NTD contribute to the immune evasion of BA.2.87.1 (Fig. 1d).

In summary, our study underscores the dynamic nature of SARS-CoV-2 evolution, particularly with the Omicron sub-lineages and emerging subvariants such as JN.1 and BA.2.87.1, which exhibit significant genetic divergence and enhanced escape capabilities. This situation calls for ongoing modifications in vaccine strategies. Updated vaccine formulations, such as SCTV01E we previously reported and WSK-V102C investigated in this study, demonstrate promising efficacy in generating high neutralizing titers against a spectrum of variants, including those with notable escape mutations. These findings highlight the crucial need for continuous monitoring and updating of vaccine formulations to keep pace with the rapidly evolving virus.

### Supplementary information

Supplemental Material

## Data Availability

All the data are provided in the main figure.
