# Supplementary material for: Robust neutralization of SARS-CoV-2 variants including JN.1 and BA.2.87.1 by trivalent XBB vaccine-induced antibodies
Source: Signal Transduct Target Ther. 2024 May 9;9:123. doi: 10.1038/s41392-024-01849-6 (PMC11082144; doi:10.1038/s41392-024-01849-6)
Supplement: Supplementary file 1 — Supplemental Material [file 41392_2024_1849_MOESM1_ESM.docx]

**Supplementary Materials**

**Robust neutralization of SARS-CoV-2 variants including JN.1 and BA.2.87.1 by trivalent XBB vaccine-induced antibodies**

Xun Wang^1^, Shujun Jiang^2^, Wentai Ma^3^, Yanliang Zhang^2^ and Pengfei Wang^1^

^1^Shanghai Pudong Hospital, Fudan University Pudong Medical Center, State Key Laboratory of Genetic Engineering, MOE Engineering Research Center of Gene Technology, School of Life Sciences, Shanghai Institute of Infectious Disease and Biosecurity, Fudan University, Shanghai, China;

^2^Department of Infectious Diseases, Nanjing Hospital of Chinese Medicine Affiliated to Nanjing University of Chinese Medicine, Nanjing Research Center for Infectious Diseases of Integrated Traditional Chinese and Western Medicine, Nanjing, Jiangsu, China;

^3^Beijing Institute of Genomics, Chinese Academy of Sciences, University of Chinese Academy of Sciences and China National Center for Bioinformation, Beijing, China.

Correspondence: Yanliang Zhang ([fsyy00404@njucm.edu.cn](mailto:fsyy00404@njucm.edu.cn)) or Pengfei Wang ([pengfei_wang@fudan.edu.cn](mailto:pengfei_wang@fudan.edu.cn))

These authors contributed equally: Xun Wang, Shujun Jiang, Wentai Ma

**MATERIALS AND METHODS**

***Serum samples***

Blood samples from two groups of individuals who had previously experienced BA.5/BF.7 breakthrough infection following three doses of inactivated vaccines were collected at the Nanjing Hospital of Chinese Medicine. One group (n=20) experienced XBB reinfection, while the other (n=11) received the Trivalent XBB Vaccine (WSK-V102C). For all COVID-19 participants, the clinical diagnosis criteria were based on the ninth National COVID-19 guidelines. All participants involved in this study had mild symptoms. All the participants provided written informed consents. All collections were conducted according to the guidelines of the Declaration of Helsinki and approved by the ethical committee of Nanjing Hospital of Chinese Medicine Affiliated to Nanjing University of Chinese Medicine (number KY2023073).

***Cell lines***

Expi293F cells (Thermo Fisher Cat# A14527) were cultured in the serum-free SMM 293-TI medium (Sino Biological Inc.) at 37 °C with 8% CO_2_ on an orbital shaker platform. HEK293T cells (Cat# CRL-3216), Vero E6 cells (cat# CRL-1586) were obtained from ATCC and cultured in 10% fetal bovine serum (FBS, GIBCO cat# 16140071) supplemented Dulbecco’s Modified Eagle Medium (DMEM, ATCC cat# 30-2002) at 37 °C, 5% CO_2_. I1 mouse hybridoma cells (ATCC, cat# CRL-2700) were cultured in Eagle’s Minimum Essential Medium (EMEM, ATCC cat# 30-2003) with 20% FBS.

***Construction and production of variant pseudoviruses***

Plasmids encoding the WT (D614G) SARS-CoV-2 spike and Omicron sub-lineage spikes, as well as the spikes with single or combined mutations were constructed. HEK293T cells were transfection with the indicated spike gene using Polyethylenimine (Polyscience). Cells were cultured overnight at 37°C with 5% CO_2_ and VSV-G pseudo-typed ΔG-luciferase (G*ΔG-luciferase, Kerafast) was used to infect the cells in DMEM at a multiplicity of infection of 5 for 4 h before washing the cells with 1×DPBS three times. The next day, the transfection supernatant was collected and clarified by centrifugation at 3000g for 10 min. Each viral stock was then incubated with 20% I1 hybridoma (anti-VSV-G; ATCC, CRL-2700) supernatant for 1 h at 37 °C to neutralize the contaminating VSV-G pseudotyped ΔG-luciferase virus before measuring titers and making aliquots to be stored at −80 °C.

***Pseudovirus neutralization assays***

Neutralization assays were performed by incubating pseudoviruses with serial dilutions of monoclonal antibodies or sera, and scored by the reduction in luciferase gene expression. In brief, Vero E6 cells were seeded in a 96-well plate at a concentration of 2×10^4^ cells per well. Pseudoviruses were incubated the next day with serially diluted samples tested in triplicate for 30 min at 37 °C. The mixture was added to cultured cells and incubated for an additional 24 h. The luminescence was measured by Luciferase Assay System (Beyotime). IC_50_ was defined as the dilution at which the relative light units were reduced by 50% compared with the virus control wells (virus + cells) after subtraction of the background in the control groups with cells only. The IC_50_ values were calculated using nonlinear regression in GraphPad Prism.

***Quantitative and statistical analysis***

The statistical analyses for the pseudovirus neutralization assessments were performed using GraphPad Prism for calculation of mean value for each data point. Each specimen was tested in triplicate. Antibody neutralization IC_50_ values were calculated using a five-parameter dose-response curve in GraphPad Prism. For comparing the serum neutralization titers, statistical analysis was performed using Multiple Mann-Whitney tests. Two-tailed p values are reported. No statistical methods were used to determine whether the data met assumptions of the statistical approach.
